# Differentiation of Dental Pulp Stem Cells on Gutta-Percha Scaffolds

**DOI:** 10.3390/polym8050193

**Published:** 2016-05-13

**Authors:** Liudi Zhang, Yingjie Yu, Christopher Joubert, George Bruder, Ying Liu, Chung-Chueh Chang, Marcia Simon, Stephen G. Walker, Miriam Rafailovich

**Affiliations:** 1 Department of Materials Science and Engineering, Stony Brook University, Stony Brook, NY 11794, USA; zldzld266@yahoo.com (L.Z.); yuyingjie312@gmail.com (Y.Y.); 2 Department of Endodontics, School of Dental Medicine, Stony Brook University, Stony Brook, NY 11794, USA; christopher.joubert@gmail.com; 3 Department of Restorative Dentistry and Biomaterials Sciences, Harvard School of Dental Medicine, Harvard University, Boston, MA 02115, USA; George_Bruder@hsdm.harvard.edu; 4 Advanced Energy Research & Technology Center, Stony Brook University, Stony Brook, NY 11794, USA; Ying.Liu.1@stonybrook.edu (Y.L.); chung-chueh.chang@stonybrook.edu (C.-C.C.); 5 Department of Oral Biology and Pathology, School of Dental Medicine, Stony Brook University, Stony Brook, NY 11794, USA; Marcia.Simon@stonybrookmedicine.edu (M.S.); Stephen.Walker@stonybrookmedicine.edu (S.G.W.)

**Keywords:** biomaterials, polymer nanocomposite, stem cells, cell differentiation, regeneration, cell-matrix interaction

## Abstract

Advances in treatment of tooth injury have shown that tooth regeneration from the pulp was a viable alternative of root canal therapy. In this study, we demonstrated that Gutta-percha, nanocomposites primarily used for obturation of the canal, are not cytotoxic and can induce differentiation of dental pulp stem cells (DPSC) in the absence of soluble mediators. Flat scaffolds were obtained by spin coating Si wafers with three Gutta-percha compounds: GuttaCore™, ProTaper™, and Lexicon™. The images of annealed surfaces showed that the nanoparticles were encapsulated, forming surfaces with root mean square (RMS) roughness of 136–211 nm. Then, by culturing DPSC on these substrates we found that after some initial difficulty in adhesion, confluent tissues were formed after 21 days. Imaging of the polyisoprene (PI) surfaces showed that biomineral deposition only occurred when dexamethasone was present in the media. Spectra obtained from the minerals was consistent with that of hydroxyapatite (HA). In contrast, HA deposition was observed on all Gutta-percha scaffolds regardless of the presence or absence of dexamethasone, implying that surface roughness may be an enabling factor in the differentiation process. These results indicate that Gutta-percha nanocomposites may be good candidates for pulp regeneration therapy.

## 1. Introduction

Root canal therapy is the most common endodontic procedure for preserving teeth without invasive surgery or dental implants [1,2]. It involves the removal of infected dental pulp, the subsequent shaping, cleaning of the hollows, and the obturation of the decontaminated canals with inert fillings such as Gutta-percha [3]. However, root canal treatment does not restore the biological function of the dental pulp tissue after significant damage [4,5]. Thus, it would be of great clinical benefit to develop a biologically based treatment to repair and regenerate the traumatized teeth, in order to both avoid surgical procedures and preserve the natural tooth.

Recently, it has been shown that in children, where the injured tooth is permanent but immature, regeneration may be an attractive alternative treatment to obturation [6,7,8]. Obturation only maintains tooth structure, while the regeneration treatment aims to allow root elongation and thickening and thereby to restore full tooth function [9]. In this case, bleeding of the pulp is induced to promote angiogenesis and pulpal regeneration with various cells including fibroblasts and odontoblast progenitors or stem cells. Among human stem cells, dental pulp stem cells (DPSC) have relatively easy accessibility, fast multiplication rate, and multi-potential capability, thus could be used in clinic applications [10,11]. It has been reported that DPSC have possibility to repair bone defect in humans [12,13]. Therefore, as undifferentiated mesenchymal stem cells, DPSC may be good candidates to study stem cell regenerative therapies.

Scaffolds may also be inserted to further activate and support the requisite stem cells of the pulp. There have been many reports on potential scaffolds for dental pulp regeneration, based on their ability to promote *in vivo* and *in vitro* DPSC differentiation with additional chemical inducers [14,15,16]. However, the use of chemical inducers such as dexamethasone corticosteroid *in vivo* may cause adverse side effects and weaken human immune system [17]. Recently Chang *et al.* have shown that polybutadiene, a polymer with similar chemistry to polyisoprene (PI), the base material of Gutta-percha, was able to induce differentiation of DPSC when the mechanical properties were properly adjusted [18]. Here we aim to explore whether Gutta-percha materials can also be appropriate scaffolds for the delivery and differentiation of DPSC without additional induce factors.

Gutta-percha is a *trans*-1,4-polyisoprene based nanocomposite, where the average molecular weight of the PI matrix was found to be 42,000 [19]. Because of the low glass transition temperature of PI, *T*_g_ = −40 °C, inorganic fillers are added to reinforce the mechanical properties [20,21]. Since bacterial infection is always a concern in root canal therapy, Zinc Oxide (ZnO) is often the particle of choice due to its well-known anti-microbial properties [22,23,24]. However, it has been demonstrated by several groups that ZnO nanoparticles are cytotoxic to eukaryotic cells [25,26,27]. Leakage of ZnO nanoparticles was suspected as an early source of cytotoxicity which became apparent after 24 h incubation of fourteen Gutta-percha materials commercially available prior to 1990 [28]. More recently though, Gambarini *et al.* showed no cytotoxicity up to 72 h to periodontal ligament cells of newer root canal filling materials [29]. No study has addressed as yet the influence of Gutta-percha on stem cells differentiation. Furthermore, if low level ZnO leakage remains a problem, it is important to determine cytotoxicity in culture for at least 21 days, or the time known to affect differentiation and biomineralization of DPSC.

In this paper, we address this question by using three types of Gutta-percha: GuttaCore™, ProTaper™, and Lexicon™. Flat scaffolds were made by spin coating Si wafers with Gutta-percha nanocomposites to facilitate imaging. Even though these scaffolds are for *in vitro* work only, they are useful for determining properties relevant to *in vivo* situation. In order to investigate their potential for supporting tooth regeneration, we first characterize the nanoparticles and their distribution using scanning electron microscopy/energy dispersive analysis X-ray spectroscopy and scanning probe microscopy. We then probe the anti-bacterial properties and the response of DPSC cultured on these scaffolds for 21 days with and without dexamethasone.

## 2. Materials and Methods

### 2.1. Materials

GuttaCore™, ProTaper™, and Lexicon™ (all from DENTSPLY International, Inc., Johnson City, TN, USA) were used in this study. ProTaper™ and Lexicon™ are uncrosslinked regular Gutta-percha cones, while GuttaCore™ includes crosslinked grey core and uncrosslinked pink coating. Inside core part of GuttaCore™ was carefully removed. All these three materials consist of an elastomeric polymer matrix, PI, loaded with inorganic nanoparticles to provide mechanical properties and radiopacity. These materials differ in their degree of loading, composition, and size of the nanoparticles. As manufacturer’s specification, they all contain 20%–30% PI matrix and more than 50% ZnO nanoparticles.

### 2.2. Preparation of Gutta-Percha Scaffolds

Silicon wafers (orientation (100), Wafer World, West Palm Beach, FL, USA), were cleaved into 1 cm × 1 cm square, boiled in a mixed ammonia-peroxide solution (H_2_O/H_2_O_2_/NH_3_H_2_O 5:1:1 by volume) for 15 min, and then boiled in piranha solution (H_2_O/H_2_O_2_/H_2_SO_4_ 3:1:1 by volume) for 15 min. They were rinsed with deionized water, and immersed in hydrofluoric acid solution (H_2_O/HF 10:1) for 30 s, to create a hydrophobic surface. ProTaper™, Lexicon™, and outside coating part of GuttaCore™ were dissolved in chloroform, and PI (*M*_w_ = 803,000 Da, polydispersity index (PDI) = 1.36, Scientific Polymer Products, Ontario, NY, USA) was dissolved in toluene. All the solutions were spun cast onto hydrophobic Si wafers at 2500 rpm for 30 s, and then annealed at 130 °C in a vacuum of 10^−3^ Torr overnight to remove the residual solvent, sterilize, and relax strains induced by the spinning process.

### 2.3. Scanning Electron Microscopy and Energy Dispersive Analysis X-ray (SEM-EDAX)

The spun cast Gutta-percha scaffolds were sputtered with gold, and imaged with a LEO/Zeiss 1550 field emission SEM (Carl Zeiss, Thornwood, NY, USA) at 20 KeV accelerating voltage using Robinson type backscattering electron detectors. The elemental compositions of the samples were determined using Phoenix EDAX system in conjunction with SEM.

### 2.4. Scanning Probe Microscopy (SPM)

Surfaces of the spun cast Gutta-percha scaffolds were assessed Bruker Dimension ICON SPM (Dimension 3000; SPM, Santa Barbara, CA, USA) scans. Both topographic (vertical scanning) and friction (lateral scanning) modes of SPM were used in this study.

### 2.5. Antimicrobial Activity and Efficacy Test 

Ten μL *Enterococcus faecalis* ATCC 19433 was sandwiched between two layers of 38 mm × 38 mm spun cast scaffolds or 29 mm diameter molded scaffolds, and incubated for 24 h. The surfaces were then rinsed to remove bacteria and the rinse solution was plated to determine colony forming units. The number of live bacterial colonies was counted at the following day.

### 2.6. Cell Isolation and Cell Plating

Human DPSC (strain AX3, Passage 6) were obtained from the Department of Oral Biology and Pathology, Stony Brook University, Stony Brook, NY, USA. They were isolated from the third molar teeth (IRB#20076778) as previously described [30] and cultured in “base media”: alpha Minimal Essential Medium (αMEM; Catalog #12571, GIBCO, Invitrogen, Carlsbad, CA, USA) supplemented with 10% fetal bovine serum (GIBCO, Invitrogen, Carlsbad, CA, USA), 100 units/mL penicillin/100 µg/mL streptomycin (GIBCO, Invitrogen, Carlsbad, CA, USA), 200 µM L-ascorbic acid 2-phosphate (Sigma Aldrich, St. Louis, MO, USA), and 10 mM β-glycerol phosphate (Sigma Aldrich, St. Louis, MO, USA). Osteogenic/odontogenic induction of the DPSC was achieved by addition of 10^−8^ M dexamethasone (Dex) (Sigma Aldrich, St. Louis, MO, USA) and is termed “induction medium” [31]. Cells were grown in a humidified incubator at 37 °C with 5% (*v*/*v*) CO_2_. Culture medium was refreshed every alternate day.

DPSC were seeded onto Gutta-percha and PI scaffolds at a density of 10,000 cells/cm^2^. The proliferation rates of DPSC grown in “base media” were determined on days 3, 6, 9, 12, and 15 by harvesting cells with Trypsin/EDTA (0.05%/0.1% in Hank’s Balanced Salt Solution) and counting cells using a hemacytometer (Hausser Scientific, Horsham, PA, USA). We didn’t specific the dead cells that may be attached to the scaffolds since they were also from proliferation. For the differentiation experiments, cells were cultured in both “base medium” and “induction medium”. For detection of mineralization by SEM-EDAX, at day 21, cultures were taken out of incubator, washed with deionized water to remove salts, and dehydrated at room temperature.

### 2.7. Confocal Laser Scanning Microscopy

Surfaces with DPSC were washed with phosphate buffered saline (pH 7.4, Ca, Mg-free), fixed with 3.7% (*w*/*v*) formaldehyde, permeabilized with 0.4% Triton X100, and then stained with Alexa Fluor 488 Phalloidin (Molecular Probes, Eugene, OR, USA) for actin filaments and Propidium Iodide (Molecular Probes, Eugene, OR, USA) for nuclei. Samples were photographed using a Leica TCS SP2 laser scanning confocal microscope (Leica microsystem Inc., Bannockburn, IL, USA) to visualize actin cytoskeleton organization and morphology of the cells.

## 3. Results

**Figure 1:**
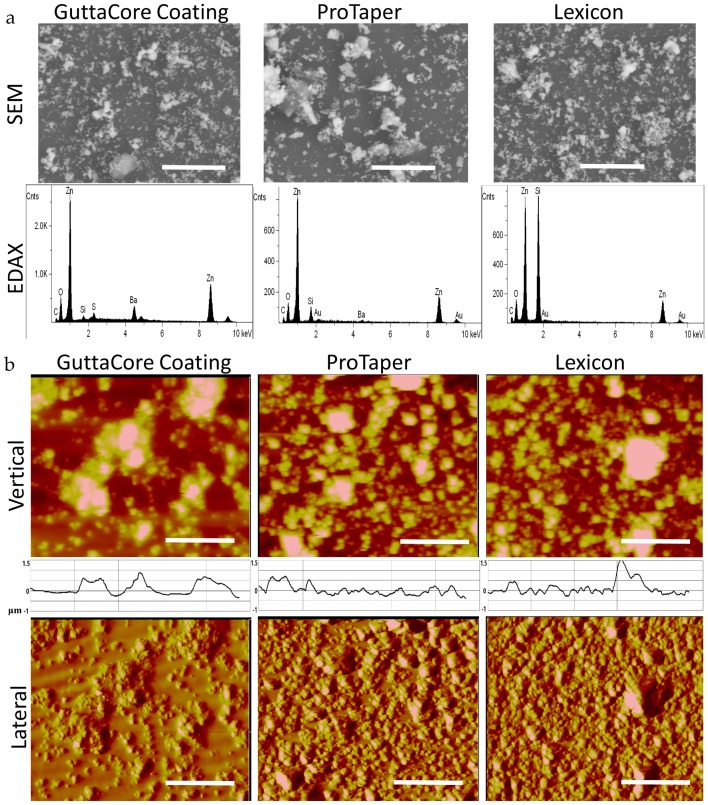
(**a**) Scanning Electron Microscopy (SEM) (**top**) images and Energy Dispersive Analysis X-ray (EDAX) (**bottom**) spectra obtained of the spun cast GuttaCore™ Coating, ProTaper™, and Lexicon™ thin (~ 1 µm) film surfaces. Scale bar is 5 μm; (**b**) Scanning Probe Microscopy (SPM) images in the topographical mode (**top**) and lateral force or friction mode (**bottom**) of the same surfaces as Figure 1a. Scale bar is 5 μm. Inset: One of the six cross sectional scans of the topographical images from which the root mean square (RMS) roughness was calculated. The Y-axis represents surface depth (−1~1.5 μm).

Gutta-percha scaffolds were obtained by dissolving ProTaper™, Lexicon™, and the outside coating of GuttaCore™ in chloroform and spin casting them onto Si wafers as thin films (*t* ~ 1 µm). SEM-EDAX analysis of the films is shown in Figure 1a where we can see that all the samples has a high density of nanoparticles. The composition of the nanoparticles is primarily ZnO, while GuttaCore™ coating also has a significant Barium component. In Figure 1b we image these surfaces with SPM, where the topographical images show particles embedded in polymer matrix, with root mean square (RMS) roughness of 186 ± 22 nm, 136 ± 8 nm, and 211 ± 30 nm for the GuttaCore™ Coating, ProTaper™, and Lexicon™, respectively. The distribution of the topographical features is consistent with that observed with SEM, namely particles are approximately 1 µm in height and are packed closely together. Moreover, we show the lateral force scans of the surfaces in Figure 1b. These scans measure the lateral deflection of the SPM tip, which is an indicator of surface friction or tip adhesion. Since the PI matrix is much softer, its friction is expected to be higher than that of the much harder particles. Yet, the relative lack of contrast observed indicates that the particles are coated by the surrounding matrix. Therefore, our hypothesis is that Gutta-percha scaffolds won’t exhibit anti-bacterial activity and cytotoxicity due to encapsulation of the ZnO nanoparticles.

**Figure 2:**
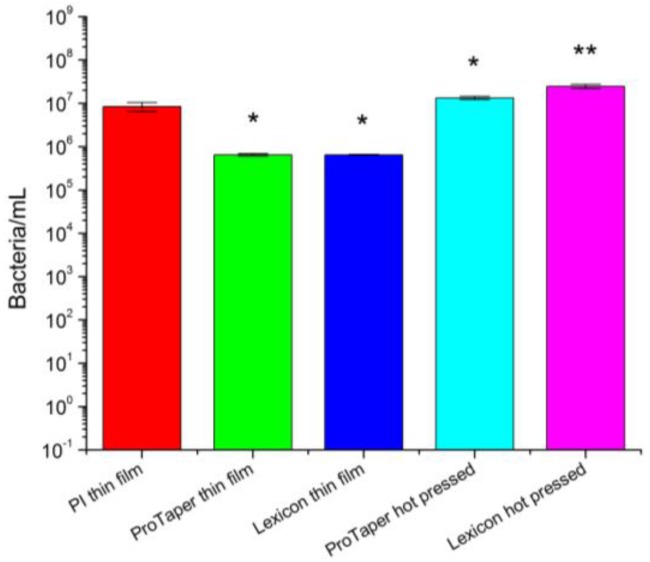
Antimicrobial activity and efficacy on ProTaper™ and Lexicon™, which were both spun cast on Si substrates and molded. A quantity of 25 nm polyisoprene (PI) coated Si substrate was used as control. The *Enterococcus faecalis* concentration applied to the surfaces was 1E7 per mL. The figure represents the number of colony forming units recovered from the scaffolds after 24 h of exposure. The results differ from control at a statistical level of *p* > 0.05 (*) and *p* < 0.05 (**).

Antimicrobial activity and efficacy test was performed to investigate the anti-bacterial properties of Gutta-percha scaffolds. Both spun cast and molded bulk samples were studied in order to eliminate possible artifacts in particle coating or distribution produced by spin casting process. PI spun coated Si wafer was used as control. The results are plotted in Figure 2. We can see that bacteria remained after 24 h incubation on molded scaffolds are almost at the same level as the beginning, while that on spun cast scaffolds are a little bit lower possibly due to the exposure of ZnO nanoparticles. In JIS Z 2801, the calculation of the value of antimicrobial activity is:

*R* = [log (*B*/*A*) − log (*C*/*A*)] (1)

where, *R*: value of antimicrobial activity. *A*: average of the number of viable cells of bacteria immediately after inoculation on the untreated test piece. *B*: average of the number of viable cells of bacteria on the untreated test piece after 24 h. *C:* average of the number of viable cells of bacteria on the antimicrobial test piece after 24 h.

According to the standard, the value of antimicrobial activity shall not be less than 2.0 for the antimicrobial efficacy. However, the value of all the tested Gutta-percha substrates is less than 2.0. Thus, none of the samples had significant anti-bacterial activity, which is consistent with previous reports [32,33].

**Figure 3:**
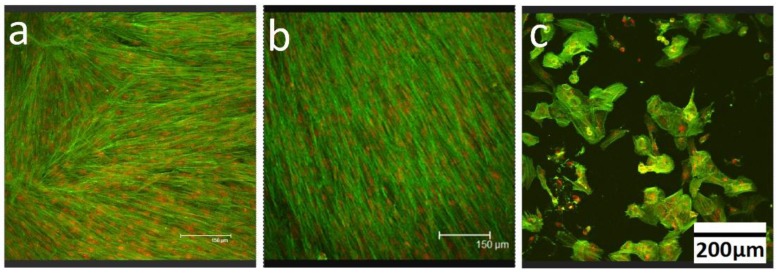
Dental pulp stem cells (DPSC) were plated on tissue culture plastic at a density of 10,000 cells/cm^2^, stained with Alexa Flour 488 (green) for actin filaments and Propidium Iodide (red) for nucleus and imaged with confocal microscopy after being cultured for (**a**) 4 days; (**b**) 6 days; and (**c**) 6 days with 0.05 mg/mL ZnO nanoparticles added at day 5.

In order to determine whether the ZnO nanoparticles alone are cytotoxic, DPSC were plated on tissue culture plastic dishes for four days, till confluence was reached (see Figure 3a). On day 5, 0.05 mg/mL of ZnO nanoparticles were added to the media [26] and the cells were cultured for another 24 h, after which they were fixed and stained with Alexa Fluor 488 Phalloidin and Propidium Iodide. The cells were then imaged with confocal microscopy, where many appear to be died, filling the plate with debris, and the remaining appears dendritic (Figure 3c). No change in the control sample was observed at day 6 (Figure 3b). These results showed that adding as low as 0.05 mg/mL of bare ZnO nanoparticles in media will prevent the normal proliferation process of cells, indicating that they are very cytotoxic to the DPSC.

**Figure 4:**
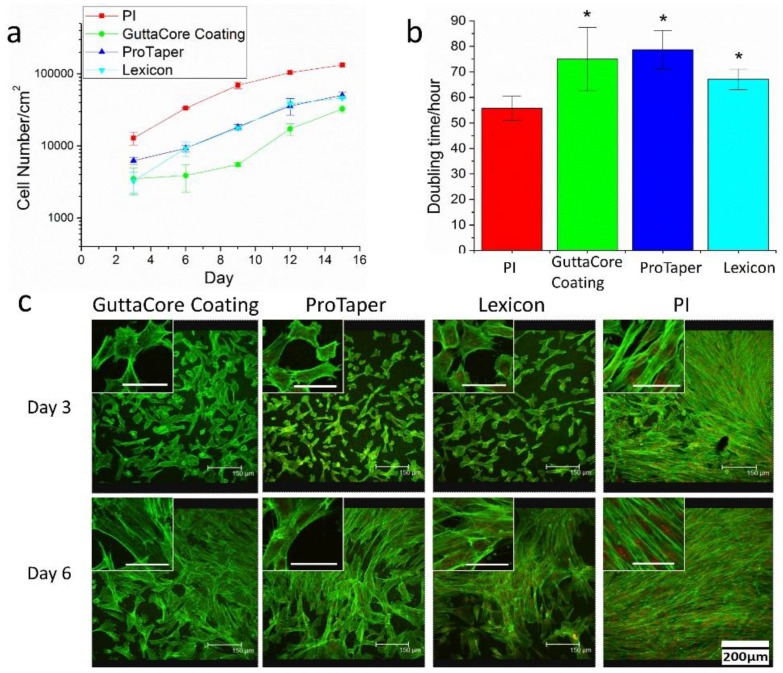
(**a**) Proliferation curves of DPSC plated, at an initial cell density of 10,000/cm^2^ on the Gutta-percha scaffolds (shown in Figure 1) and 25 nm spun cast PI thin films; (**b**) The doubling time of the DPSC calculated from the data plotted in (**a**). The Gutta-percha samples differ from control sample at a statistical level of *p* > 0.05 (*); (**c**) Confocal microscopy images of DPSC plated on PI and on the three Gutta-percha scaffolds after 3 and 6 days in culture without dexamethasone. Actin filaments were stained with Alex Flour 488 (green) and nucleus was stained with propidium iodide (red). Inset: High magnification images of individual cells in the cultures without dexamethasone (scale bar is 50 μm).

Then, spun cast Gutta-percha scaffolds, together with 25 nm PI thin films, were used to probe whether the ZnO nanoparticles in Gutta-percha are cytotoxic. DPSC were seeded at a density of 10,000/cm^2^ onto the scaffolds and cell proliferation rates were measured as a function of incubation time. The cell proliferation curves and doubling time calculations are shown in Figure 4a,b. From the figure we find that comparing to the PI control, the proliferation curves of the Gutta-percha scaffolds first show a lag where the proliferation rate is slower, but then the culture recovers and proliferates at nearly the same rate as the control. This result is consistent with the confocal microscopy images obtained at days 3 and 6 in Figure 4c. It is obviously that at day 3, the DPSC only formed a confluent sheet on the PI control sample, but not on any of the Gutta-percha scaffolds. In the inset of each figure we show a magnified view of the cells, where we can clearly see that the actin fibers are well extended only on PI. On the Gutta-percha scaffolds, the fibers are thin and not well extended. The cell areas are also much smaller. However, by day 6, the cells on all the Gutta-percha scaffolds appear to have recovered, and formed a confluent sheet. The morphology of the individual cells shown in the inset now appears similar to that of the cells on the control sample with well-defined actin fibers (Figure 4c, inset). This observation of Gutta-percha scaffolds demonstrates the lack of cytotoxicity towards ligament cells, together with the decreased anti-bacterial activity, support the hypothesis of encapsulation.

**Figure 5:**
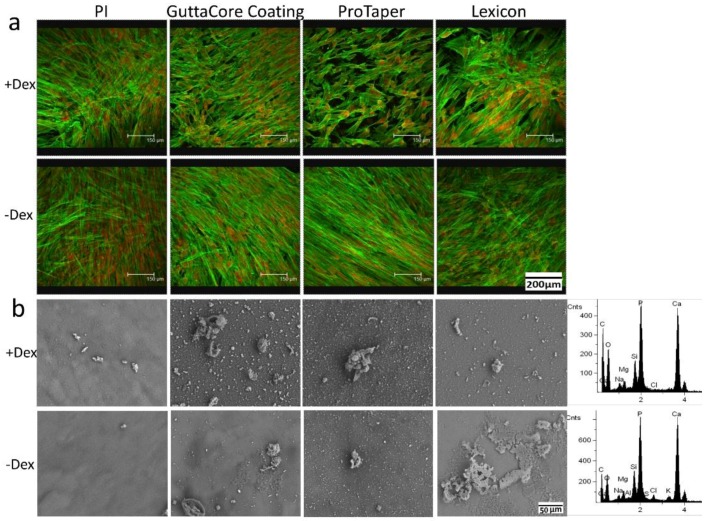
(**a**) Confocal microscopy images of the DPSC plated on scaffolds the same as Figure 4 and cultured for 21 days with (**top**) and without (**bottom**) addition of dexamethasone. Actin filaments were stained with Alex Flour 488 (green) and nucleus was stained with propidium iodide (red); (**b**) SEM images of the surfaces after rinsing with DI water and dehydration in air. The sidebar shows typical EDAX spectra obtained from the mineral deposits in the cultures with dexamethasone (**top**) and without dexamethasone (**bottom**).

To further explore the potential application of Gutta-percha on pulp regeneration, the ability of DPSC to differentiate on these scaffolds was also probed. Cells were incubated with and without dexamethasone which is known to induce osteogenic/odontogenic differentiation of DPSC [31,34]. After 21 days in culture, the samples were imaged with confocal microscopy and examined using SEM-EDAX. From Figure 5a we can see that all cultures remained confluent for at least 21 days, indicating that there was no slow leakage of ZnO nanoparticles sufficient to be cytotoxic. The cultures with added dexamethasone produced biomineralized deposits, which EDAX confirmed as being composed of calcium phosphate, or hydroxyapatite (HA) (Figure 5b). Hence Gutta-percha scaffolds did not interfere with the standard differentiation process. On the other hand, it’s interesting to note that differentiation, as defined by deposition of biomineralized HA products, was also observed on all of the Gutta-percha scaffolds without dexamethasone. Hence we can conclude that Gutta-percha is neither anti-bacterial nor cytotoxic and may promote DPSC differentiation in the absence of additional chemical factors.

## 4. Discussion

Gutta-percha materials were primarily designed for obturation of the canal. Consequently, they were mostly composed of an elastomeric polymer mixed with ZnO nanoparticles, whose function were primarily to provide anti-bacterial protection and mechanical reinforcement. Hence little attention was paid to their interaction with living tissues. More recently new therapies have been proposed, especially in children and adolescents, where injury to the tooth could be treated via regeneration of the pulp rather than obturation of the canal. Here we show that the same Gutta-percha materials used for obturation may also be suitable for tooth regeneration despite their large volume fraction of ZnO nanoparticles, which as expected, were shown to be cytotoxic when added directly into the culture media. Scaffolds obtained from the Gutta-percha materials maintained encapsulation of the ZnO nanoparticles within the polymer matrix. Although cells plated on these substrates initially showed lower plating efficiency and proliferation rate, after six days, the culture seemed to have recovered and the cells grew to confluence with a doubling time similar to that on the PI homopolymer.

**Figure 6:**
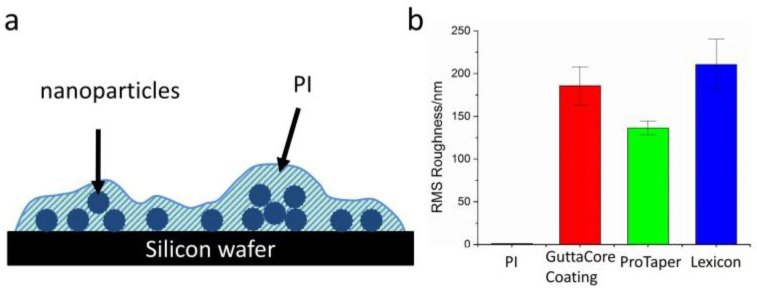
(**a**) Schematic illustration of a typical Gutta-percha nanocomposite scaffolds; (**b**) RMS roughness of PI, GuttaCore™ Coating, ProTaper™ and Lexicon™ samples calculated from the SPM topographical images. Note the value for PI, 1.2 ± 0.1 nm is too small to appear clearly.

DPSC were also cultured on Gutta-percha and PI scaffolds for 21 days with and without dexamethasone, a soluble factor known to induce osteogenic/odontogenic differentiation. Analysis of the PI surface after 21 days showed biomineralized deposits only on the cultures treated with dexamethasone. The DPSC cultured on the Gutta-percha scaffolds with dexamethasone also showed biomineralized deposits indicating that the ZnO nanoparticles did not interfere with the cell culture and standard cell differentiation even after three weeks. More interesting though was the observation of biomineralized deposits on the scaffolds where the cells were cultured without dexamethasone, indicating that those scaffolds were able to induce differentiation without additional chemical factors. The high toxicity of the bare ZnO nanoparticles and the fact that the DPSC were able to form a confluent sheet of tissue after 21 days precludes significant leakage of ZnO into the culture media. Hence we could make the model shown in Figure 6 of the Gutta-percha scaffolds, where large amounts of ZnO and/or Barium particles are fully encapsulated within the PI matrix. The major distinction then between the Gutta-percha samples and the control is the surface roughness measured with SPM topographical scans. Recently, Iaculli Flavia demonstrated that the probability of osteogenic differentiation of DPSC without chemical induction factors was increased on rough Titanium surfaces [35]. A similar phenomenon may also be occurring here, where the differentiation is induced simply by the surface roughness. In fact, the largest amount of HA, where the deposition has formed sheet like deposits is observed on the Lexicon™ scaffold, which also has the largest RMS roughness of 211 ± 30 nm, and while the least amount is observed on the ProTaper™ with an RMS roughness of 136 ± 8 nm.

## 5. Conclusions

In this study, we have demonstrated that the ZnO nanoparticles in the scaffolds obtained from Gutta-percha nanocomposites were encapsulated within the polymer matrix. No significant anti-bacterial or cytotoxic effects could be determined. DPSC plated on these scaffolds were shown to proliferate and differentiate without additional soluble factors after 21-day incubation. Hence the mechanical strength and roughness imparted by the nanoparticles contributed to promoting differentiation of DPSC placed in contact with the material surfaces. These results indicate that in addition to obturation, Gutta-percha nanocomposites ProTaper^TM^, Lexicon^TM^, and GuttaCore^TM^ may also be used as scaffolds for tooth regeneration.
